# Metallurgy and Chemistry of Metals

**Published:** 1879-12

**Authors:** 


In the report of section VI, Metallurgy and Chemistry of the Metals, the committee say:

"The section desires to present at this time to the Association three conclusions:

1.—That pure gold introduced into a tooth as a filling is inert and *of itself* exercises no electric or galvanic action on the tooth.

2.—That while amalgam fillings conform to the general shape of the cavities, the surfaces of the fillings do not fit closely to the surfaces of the cavities, but are rough and uneven or indented, the indented or depressed portions not coming in contact with the dentine, thus being liable to leak, and in fact a very large number do leak.

3.—That plastic fillings into the composition of which chlorine enters, cannot be relied upon as permanent; first, because of the great affinity of chlorine for alkalies. Second, because the other constituents of the filling have greater affinity for other acidulous agents than for chlorine,—these affinities bringing about a disintegration of the filling."

These statements and conclusions are in broad variance with the "new departure;" and a new theory—that of *destructive alkalinity*,—broached. Will the authors of this paper please furnish the public the grounds on which they rest their belief, and the steps by which such conclusions are arrived at?

